# Strain Engineering of Cu_2_O@C_2_N for Enhanced Methane-to-Methanol Conversion

**DOI:** 10.3390/molecules30153073

**Published:** 2025-07-23

**Authors:** Shuxin Kuai, Bo Li, Jingyao Liu

**Affiliations:** 1 Institute of Theoretical Chemistry, College of Chemistry, Jilin University, Changchun 130023, China; kuaisx23@mails.jlu.edu.cn; 2 Institute of Catalysis for Energy and Environment, College of Chemistry and Chemical Engineering, Shenyang Normal University, Shenyang 110034, China

**Keywords:** methane monooxygenase, C_2_N monolayer, methane conversion, strain engineering, density functional theory

## Abstract

Inspired by the active site of methane monooxygenase, we designed a Cu_2_O cluster anchored in the six-membered nitrogen cavity of a C_2_N monolayer (Cu_2_O@C_2_N) as a stable and efficient enzyme-like catalyst. Density functional theory (DFT) calculations reveal that the bridged Cu-O-Cu structure within C_2_N exhibits strong electronic coupling, which is favorable for methanol formation. Two competing mechanisms—the concerted and radical-rebound pathways—were systematically investigated, with the former being energetically preferred due to lower energy barriers and more stable intermediate states. Furthermore, strain engineering was employed to tune the geometric and electronic structure of the Cu-O-Cu site. Biaxial strain modulates the Cu-O-Cu bond angle, adsorption properties, and d-band center alignment, thereby selectively enhancing the concerted pathway. A volcano-like trend was observed between the applied strain and the methanol formation barrier, with 1% tensile strain yielding the overall energy barrier to methanol formation (ΔG_overall_) as low as 1.31 eV. N_2_O effectively regenerated the active site and demonstrated strain-responsive kinetics. The electronic descriptor Δε (ε_d_ − ε_p_) captured the structure–activity relationship, confirming the role of strain in regulating catalytic performance. This work highlights the synergy between geometric confinement and mechanical modulation, offering a rational design strategy for advanced C1 activation catalysts.

## 1. Introduction

Methane (CH_4_), the major component of natural gas, is a clean and abundant energy source characterized by high calorific value, low cost, and high safety. Owing to its wide availability, methane has emerged as a promising feedstock for producing high-value chemicals and fuels [1,2,3,4]. Methane molecules possess a high bond energy of 435 kJ·mol^−1^, which significantly hinders the activation of C-H bonds [5]. In practical applications, methane is mainly used as a fuel for heat generation. With the large-scale production of natural gas hydrates and biogas worldwide, the synthesis of liquid fuels and chemicals using methane as a raw material has garnered increasing attention [6,7,8]. CH_4_ can be selectively converted into value-added products, including methanol (CH_3_OH), methyl hydroperoxide (CH_3_OOH), formic acid (HCOOH), acrylic acid (C_3_H_4_) [9,10,11,12,13]. Among various catalytic routes, the selective oxidation of methane to methanol is one of the promising ways to be industrially implemented [14,15,16].

Various catalytic active materials have been examined for methane activation, including Metal Pt [17], Metal Oxide [18], metal-exchange zeolites (e.g., Cu-zeolite [19,20], and Fe-zeolite [21]), metal dimers embedded in phthalocyanine monolayers [22] and carbon-based materials doped with Fe [23], Zn [24], Pt [25], and Co [23]. Although great progress has been achieved, methane activation under mild conditions remains unresolved. Methane monooxygenase (MMO), an enzyme capable of selectively oxidizing methane to methanol at ambient temperature, offers valuable inspiration. The active site of this enzyme predominantly consists of iron and copper [26,27,28,29]. Molecular complexes have been designed to emulate their structure and reactivity through the versatility of ligand design. In particular, copper-oxygen complexes, including Cu_2_O_2_ [trans-1,2-peroxide, μ-η^2^-peroxide and bis(μ-oxy) dicopper cores] and Cu_2_O [mono(μ-oxy) dicopper cores], have been extensively studied, with their spectral fingerprints well-documented [30]. However, the thermal stability of these catalysts is not sufficient for application, and much effort has been devoted to identifying suitable carriers to stabilize the active center of methane monooxygenase for better stability.

The novel carbon–nitrogen porous two-dimensional C_2_N has been successfully fabricated through a straightforward bottom-up wet chemical strategy [31], and has aroused significant interest [32,33,34]. It features sp^2^ hybridized nitrogen atoms with lone-pair electrons at the pore edges, which strongly interact with the metal species, thus providing a thermally stable anchoring site for metal catalysts. Moreover, the high surface-to-volume ratio ensures the adequate exposure of active sites, which significantly facilitates the effective adsorption and activation of reactant molecules. Inspired by the mono(μ-oxo) dicopper core structure in methane monooxygenase, we sought to construct a biomimetic Cu-O-Cu motif by embedding a Cu_2_O cluster into the six-membered nitrogen cavity of C_2_N. The confined Cu-O-Cu bridge, stabilized by the N_6_ coordination environment, mimics the enzymatic active center while offering enhanced thermal stability and electronic tunability. Among nitrogen-rich carbon frameworks, graphitic carbon nitride (g-C_3_N_4_) has been widely employed as a catalyst support due to its thermal robustness (up to ≈600 °C in air), layered structure, and diverse nitrogen functionalities. It has shown promising performance in stabilizing Cu_2_O clusters for selective oxidation reactions [35]. However, g C_3_N_4_ prepared by thermal polymerization typically exhibits low crystallinity and disordered interlayer stacking, which leads to non-uniform nitrogen sites and disordered pore systems that hinder precise control over metal anchoring and coordination tuning [36]. In contrast, C_2_N features a highly ordered 2D microporous architecture composed of regularly arranged pyridinic nitrogen atoms within a conjugated carbon framework. This structure not only enhances thermal and chemical stability but also enables the formation of homogeneous and tunable metal-ligand coordination environments [37,38]. These advantages make C_2_N a compelling platform for supporting Cu_2_O clusters. Accordingly, we designed Cu_2_O@C_2_N to explore its ability to stabilize active sites and modulate reaction pathways more effectively than conventional g-C_3_N_4_-based systems.

Meanwhile, it has been proven that strain engineering is beneficial for tuning the electronic structure properties of 2D materials [39,40,41]. Strain engineering can regulate both geometry and electronic structures in a systematic manner, which can effectively adjust the catalytic performance [42,43,44,45]. The research by Fan and others has shown that strain engineering can adjust the d-band center and work function of catalysts, thereby enhancing the catalytic performance of Pt-doped Ti_2_CX_2_ (X = O and C) in oxygen reduction reactions (ORRs) and oxygen evolution reactions (OER) [46,47]. This paper further demonstrates the influence of strain on the catalytic performance of thermal catalysts through the study of the catalyst’s electronic structure and the mechanism of partial oxidation of methane to methanol.

In this study, we present the design of a novel enzyme catalyst featuring Cu_2_O anchored within the six-membered ring cavity of C_2_N. Concurrently, we modulate the catalyst’s performance via strain engineering. This work aims to develop an effective strategy for regulating the partial oxidation of methane to methanol. The calculations indicated that the applied strain successfully adjusted both the geometry and the electronic structure of the active center of Cu-O-Cu, which consequently affected the catalytic performance. The significant decrease in the overall energy barrier for methanol formation (ΔG_overall_) under applied strain demonstrates a notable strain-induced enhancement in catalytic activity. Two competing reaction mechanisms—the free radical–rebound pathway and the concerted pathway—were systematically compared under strained and unstrained conditions. In this study, the effectiveness of Cu_2_O@C_2_N as an efficient catalyst for the partial oxidation of methane to methanol was validated by simulation. Furthermore, it establishes strain engineering as a viable strategy for modulating catalytic performance in two-dimensional support systems, thereby offering new perspectives for the rational design of advanced methane activation catalysts. Together, this work not only confirms the catalytic viability of Cu_2_O@C_2_N under strain but also provides valuable insights into how electronic-geometric coupling governs C1 conversion reactivity.

## 2. Results and Discussion

### 2.1. Structural and Electronic Properties of Cu_2_O@C_2_N

**Figure 1:**
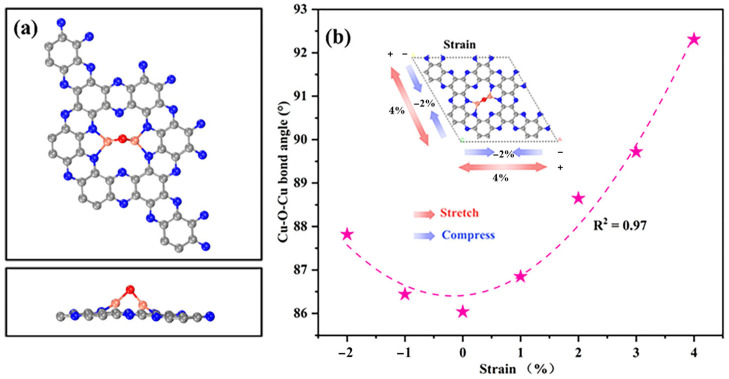
(**a**) Top and side views of the optimized structure of Cu_2_O@C_2_N. (**b**) Variation in the Cu-O-Cu bond angle under applied strain.

Inspired by methane monooxygenase, the Cu_2_O@C_2_N system was constructed to mimic its dinuclear copper-oxo active site. The optimized geometry of the Cu_2_O@C_2_N hybrid system (Figure 1a) demonstrates a characteristic bridged Cu-O-Cu configuration stabilized within the hexagonal nitrogen cavity (N_6_) of the C_2_N monolayer. Structural analysis shows Cu-O bond lengths of 1.79 Å and a Cu-O-Cu bond angle of 86.04°, forming a compact coordination environment. Notably, the intermetallic Cu-Cu distance of 2.23 Å aligns closely with previously reported values for similar copper oxide systems [48]. To investigate the strain-dependent structural evolution, biaxial strains ranging from −2% to +4% were systematically applied to the Cu_2_O@C_2_N hybrid system. As depicted in Figure 1b, both compressive and tensile strains consistently increase the Cu-O-Cu bond angle, with a +4% tensile strain causing a pronounced angular expansion to 92.31°, while a −2% compressive strain moderately enlarges the angle to 87.82°. Notably, tensile deformation exhibits superior angular distortion efficiency.

**Figure 2:**
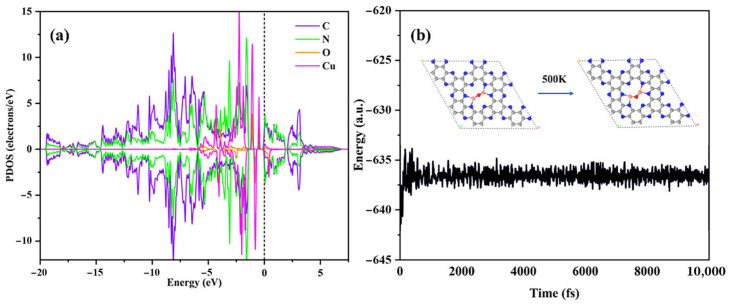
(**a**) PDOS diagram of the Cu_2_O@C_2_N catalyst without applied strain. (**b**) Schematic representation of total energy variation during AIMD simulations of Cu_2_O@C_2_N at 500 K.

Projected density of states (PDOS) analysis of the strain-free Cu_2_O@C_2_N heterostructure reveals pronounced orbital hybridization between copper d-orbitals and the C_2_N substrate (Figure 2a), indicative of strong interfacial electronic coupling [49]. Strain-modulated configurations exhibit distinct electronic coupling characteristics, as detailed in Supplementary Figure S1. The charge density difference in Figure S2 further suggests that electrons are transferred from the Cu atoms to the C_2_N bases, thus stabilizing the Cu_2_O species within the N_6_ cavity of C_2_N. Upon coordination with oxygen, additional electron transfer to the oxygen atom confirms the redox-active nature of the Cu-O-Cu center. These results underscore the role of C_2_N as a thermally and electronically stable support for anchoring Cu_2_O clusters. The thermodynamic stability of the Cu_2_O@C_2_N system under strain was assessed via formation energy calculations, all yielding negative values, indicating favorable energetics (Figure S3). To assess kinetic stability, AIMD simulations were performed at 500 K for 10 ps. To evaluate the thermodynamic and kinetic stability of the Cu_2_O@C_2_N system, we conducted formation energy calculations and ab initio molecular dynamics (AIMD) simulations. All calculated formation energies under both unstrained and strained conditions were negative, indicating favorable thermodynamics (Figure S3). AIMD simulations were performed at 500 K for 10 ps using a 1 fs time step and the Nosé–Hoover thermostat. Throughout the simulation, the Cu_2_O cluster remained stably confined within the C_2_N cavity, showing no significant diffusion. The time evolution of total energy for the unstrained system is shown in Figure 2b, while the results for −2% to +4% strain conditions are provided in Figure S4. In all cases, the small energy fluctuations confirm good thermal stability at the atomic scale, which may benefit future upscaling. To further validate the structural integrity, we analyzed the average Cu–O bond lengths during AIMD simulations. Three representative strain states were selected for analysis: compressive (−2%), unstrained (0%), and tensile (+4%). In all cases, minimal bond length fluctuations were observed (Figure S5), indicating high structural robustness under moderate mechanical perturbation. While these 10 ps simulations demonstrate short-term stability, longer timescale simulations or experimental validation would be needed for industrial applications. Overall, these results highlight the dual thermal and mechanical resilience of the Cu_2_O@C_2_N catalyst, establishing a reliable foundation for future catalytic design.

### 2.2. Catalytic Performance: Methane to Methanol Conversion

#### 2.2.1. Reaction Mechanisms: Concerted vs. Radical–Rebound

**Figure 3:**
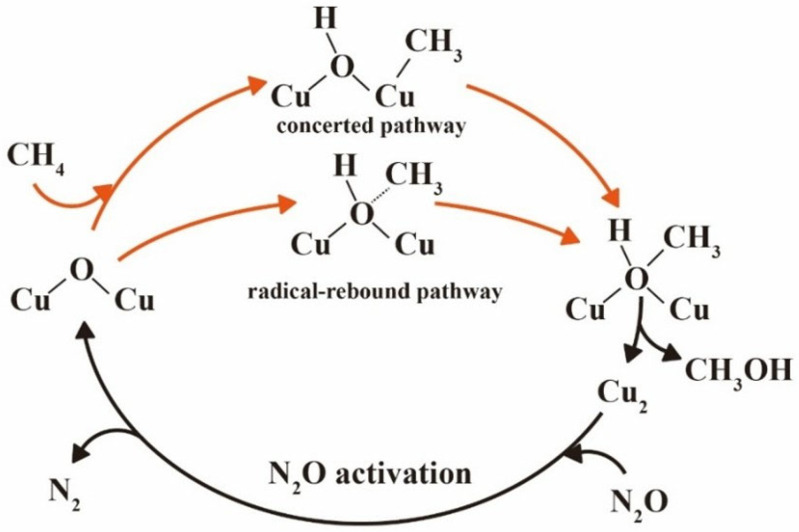
Schematic representation of the surface-mediated catalytic cycle mechanism for the partial oxidation of methane to methanol using Cu_2_O@C_2_N catalysts.

**Figure 4:**
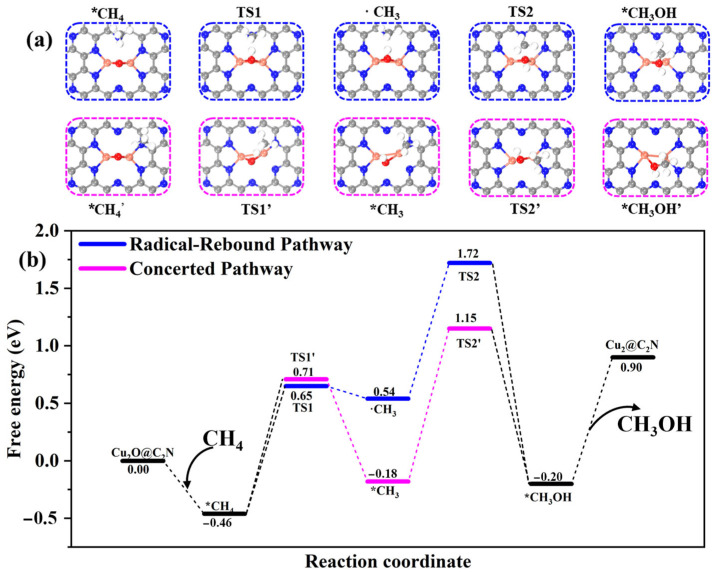
(**a**) Optimized structures of key intermediates and transition states involved in the radical-rebound (top, blue dashed boxes) and concerted (bottom, magenta dashed boxes) pathways for methane oxidation to methanol. Only the most stable configurations of CH_4_ and CH_3_OH are shown and are highlighted in black to indicate that they are common to both pathways. (**b**) Free energy profiles of methane oxidation on Cu_2_O@C_2_N via the radical-rebound (blue) and concerted (magenta) mechanisms. Energies are referenced to CH_4_ and the catalyst surface. An asterisk (*) represents adsorbed species.

To uncover the mechanistic nature of methane partial oxidation on Cu_2_O@C_2_N, two competing reaction pathways were examined: the concerted pathway and the radical–rebound pathway, both illustrated in Figure 3. In the concerted mechanism, the CH_4_ molecule is weakly adsorbed on the Cu-O-Cu active site with an adsorption energy of −0.46 eV. At this stage, no bond forms between methane and the catalyst, and the distance between the O atom and the nearest H atom in CH_4_ is 2.48 Å. As the reaction proceeds, C-H bond cleavage occurs in a single elementary step, transferring the hydrogen to the bridging oxygen and anchoring the resulting methyl group to the Cu center. The transition state exhibits a stretched C-H bond and a partially formed Cu-CH_3_ interaction, as shown in Figure 4a. The energy barrier for this C-H activation step is 1.37 eV. Subsequently, the methyl group couples to the hydroxyl group to produce methanol with a relatively low energy barrier of 1.33 eV; ΔG_overall_ equals G_TS2’_ − G_CH4’_ equals 1.61 eV, as shown in Figure 4b.

In contrast, the radical–rebound mechanism proceeds via initial homolytic cleavage of the C-H bond, forming a methyl radical and a surface OH group. The methyl radical remains weakly bound above the active site and gradually rebounds toward the hydroxyl group to form methanol, as shown in Figure 4a. The activation energy for the initial C-H scission is 1.11 eV, and the barrier for the radical coupling step is 1.18 eV. Despite the slightly lower barrier for C-H cleavage, the rebound pathway exhibits a higher ΔG_overall_ (2.18 eV), as shown in Figure 4b. Moreover, the inherent instability and high reactivity of the methyl radical render this pathway kinetically less favorable and more susceptible to side reactions such as dimerization or oxidation [50]. Overall, the concerted pathway is identified as the dominant route due to its lower energy span and greater thermodynamic and kinetic favorability. The stabilization of key intermediates via strong Cu-CH_3_ and Cu-OH interactions plays a critical role in steering the reaction toward the concerted mechanism.

#### 2.2.2. Effect of Strain on Mechanism Selectivity

**Figure 5:**
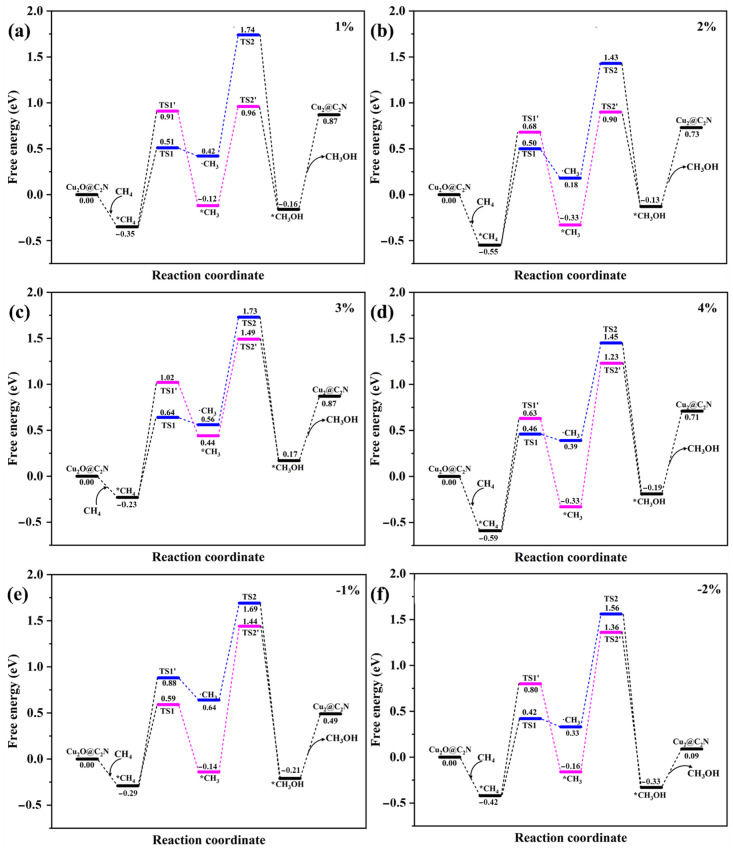
Schematic free energy diagrams for the concerted and radical–rebound pathways under different strain conditions: (**a**) 1%; (**b**) 2%; (**c**) 3%; (**d**) 4%; (**e**) −1%; (**f**) −2%. Blue traces represent the radical–rebound pathway; pink traces represent the concerted pathway.

To further elucidate the effect of strain on reaction mechanisms, we compared the energy profiles of both pathways under a range of applied strains (−2% to +4%), as summarized in Figure 5. At 0% strain, the concerted pathway exhibits a lower ΔG_overall_ (1.61 eV) than the radical-rebound route (2.18 eV), aligning with the intrinsic preference for the concerted mechanism. Upon application of 1% tensile strain, the energy barrier for the concerted path further decreases to 1.31 eV, while the rebound path shows a slightly reduced total barrier of 2.09 eV, preserving the energetic preference for the former.

To facilitate comparison, the corresponding ΔGoverall values for both pathways under each train condition are summarized in Table 1. As strain increases to 2–4%, the energy barriers of both pathways fluctuate, but the concerted mechanism consistently remains more favorable. For instance, at 2% strain, the ΔGoverall are 1.45 eV for the concerted pathway and 1.98 eV for the rebound pathway; at 3% strain, they are 1.72 eV and 1.96 eV, respectively. Even under compressive strain of −1%, the concerted pathway remains dominant, exhibiting an ΔGoverall of 1.73 eV, in contrast to 1.98 eV for the rebound mechanism. The structures of the intermediate and transition states are shown in Figures S6–S11.

This strain-dependent divergence in reaction energetics is attributed to geometric tuning of the Cu-O-Cu active site. As shown in Figure 1b, tensile strain leads to a progressive increase in the Cu-O-Cu bond angle, which enhances spatial accommodation for the transition state in the concerted path. Meanwhile, the rebound mechanism suffers from limited stabilization of the methyl radical under distorted geometries, especially at higher strains. In particular, the 1% tensile strain condition not only results in the lowest ΔG_overall_ (1.31 eV) but also offers an optimal configuration for methyl-Cu interaction, promoting efficient methanol desorption. This confirms the concerted pathway as both kinetically and geometrically favored under moderate tensile strain. These results underscore the critical role of strain engineering in modulating transition state energetics, dictating pathway selectivity, and enhancing catalytic efficiency for methane partial oxidation. We also monitored the magnetic moments along the reaction pathway. Notably, the magnetic moment at the Cu center increased from 0.26 μB to 1.68 μB during the transition from CH_4_ adsorption to CH_3_OH formation, likely due to local spin redistribution associated with adsorbate-induced electronic changes. Nevertheless, no spin crossover occurred, and the overall spin state remained consistent throughout the reaction. Our calculations indicate that the desorption energy of methanol is lower than the energy barriers required for C-H activation and C-O bond formation, suggesting that methanol can be released relatively easily from the Cu site and is unlikely to act as a persistent blocking ligand.

Notably, even under the most favorable condition (1% strain), the overall free energy barrier (ΔG_overall_) remains as high as 1.31 eV, which exceeds the threshold typically required for industrially relevant catalytic performance. To further reduce this barrier, future efforts may focus on introducing co-catalysts or dopants to modulate the local electronic structure and improve active site properties. In addition, synergistic approaches involving photocatalysis or electrocatalysis—such as harnessing photogenerated charge carriers or applying external potentials—may enhance charge transfer and intermediate activation. These directions offer promising routes to improve both the thermodynamic and kinetic aspects of the catalytic process.

Although great progress has been achieved, methane activation under mild conditions remains unresolved. Recent studies continue to explore new catalyst systems to address this challenge. For instance, Huang and Liu [51] reported efficient methane-to-methanol conversion using Cu-based materials, while Liu et al. [52] revealed a radical-mediated mechanism on CuO-rutile catalysts, emphasizing oxygen radical species and CH_3_ intermediates. These works offer valuable insights but also highlight the need for improved stability and tunability in catalyst design.

While the theoretical results highlight the catalytic advantages of strain modulation in the Cu_2_O@C_2_N system, its experimental realization remains challenging. Key difficulties include achieving accurate control of strain distribution, maintaining mechanical stability under applied strain, and ensuring synthesis uniformity. To address these challenges, several strain-engineering strategies, such as substrate-induced strain, thermal expansion mismatch, and lattice mismatch via epitaxial growth, have been widely applied to other 2D materials, and may offer viable paths for experimentally realizing strained Cu_2_O@C_2_N monolayers in future studies [53].

In summary, the applied strain significantly modulates the relative preference between the two mechanisms by tuning both the transition state geometry and the electronic interaction at the Cu-O-Cu center. The concerted pathway is consistently favored under all strain conditions, with 1% tensile strain yielding the most kinetically and electronically optimized configuration for methanol formation.

### 2.3. Active Site Regeneration and Strain-Responsive Reactivation Behavior

**Figure 6:**
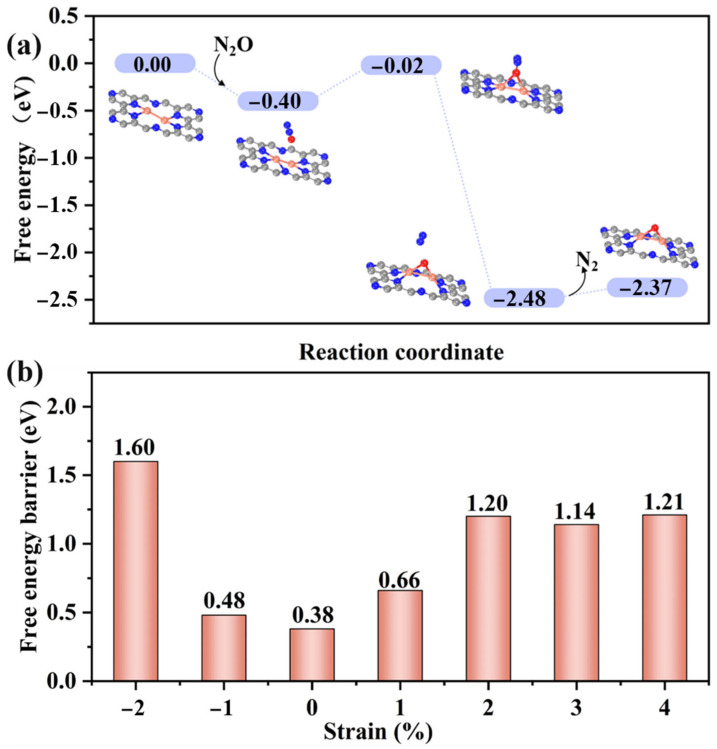
(**a**) Free energy profile and structures of intermediates for the regeneration of the Cu-O-Cu active site via N_2_O decomposition on the Cu_2_O@C_2_N catalyst. (**b**) Free energy barriers for N_2_O decomposition under various biaxial strain conditions.

For sustained catalytic performance in methane oxidation, the regeneration of the active Cu-O-Cu site after methanol desorption is essential. Here, N_2_O was chosen as the oxidant to reintroduce reactive oxygen species in order to regain the oxygen source. As shown in Figure 6a, the N_2_O molecule was initially adsorbed on the Cu_2_O@C_2_N surface with an adsorption free energy of −0.40 eV in a parallel conformation, with the oxygen terminus oriented toward the Cu dimer. During the activation process, the N-O bond elongates from 1.20 Å to 1.30 Å as the molecule approaches the active center. The transition state for N-O bond cleavage leads to N_2_ release and formation of a new Cu-O-Cu species, completing the regeneration cycle. The activation barrier for this decomposition is only 0.38 eV under unstrained conditions, indicating high efficiency.

To further assess the role of strain engineering in the regeneration process, we applied biaxial strain ranging from −2% to +4% and tracked the corresponding energy barriers (Figure 6b). The results demonstrate a non-monotonic strain dependence of reoxidation efficiency. At 1% tensile strain, the barrier for N_2_O decomposition slightly increases to 0.66 eV but remains low enough to enable facile regeneration. However, excessive tensile strain (e.g., 2–4%) leads to fluctuating or elevated barriers (e.g., 1.20 eV at 2%), suggesting geometric distortion around the Cu site weakens its oxidative reactivity.

These observations reinforce the conclusion that strain modulates not only the catalytic reaction pathways but also the regeneration dynamics. Optimal strain (~1%) ensures not only efficient methane conversion but also sustainable catalytic cycling by maintaining a favorable Cu electronic configuration and surface adsorption geometry for oxidant activation. Therefore, active-site reoxidation and catalytic turnover are intimately coupled to the strain-tuned structural and electronic flexibility of the Cu_2_O@C_2_N system. It is worth noting that although N_2_O effectively facilitates catalyst regeneration, it is also a potent greenhouse gas with a high global warming potential. However, if the N_2_O used in this context is sourced from industrial waste gases, its consumption during the regeneration process—where it is reduced to environmentally benign N_2_—could offer dual advantages: enabling catalytic reactivation while simultaneously contributing to greenhouse gas mitigation. From this perspective, N_2_O may serve not only as a regeneration agent but also as a vector for valorizing waste emissions. Alternatively, more sustainable oxidants such as molecular O_2_ may be employed, particularly through photocatalytic or electrocatalytic activation strategies that enhance charge transfer efficiency. These directions warrant further investigation to minimize environmental impact while maintaining catalytic performance.

### 2.4. Activity Origin and Electronic Descriptor Analysis

**Figure 7:**
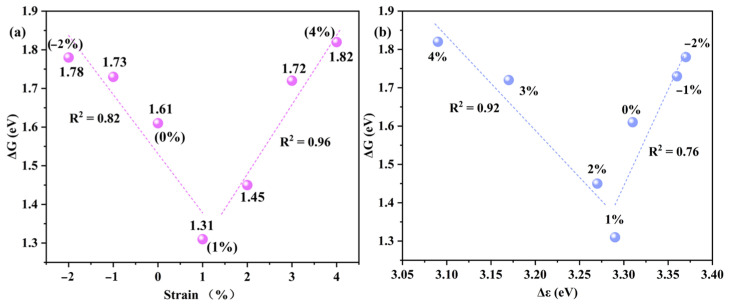
Strain-dependent free energy barriers (ΔG) for CH_4_ → CH_3_OH conversion, showing an inverse volcano relationship. (**a**) ΔG versus applied strain. (**b**) ΔG versus the Cu-O-Cu electronic descriptor (Δε) under corresponding strain conditions.

Although the regeneration step was evaluated separately, the ΔG_overall_ remains the rate-determining component in the overall cycle under most strain conditions, as shown in Figure S12. Therefore, the ΔG_overall_ for methanol formation was used as the activity descriptor to qualitatively compare the effect of strain. To reveal the electronic origin underlying the strain-dependent catalytic activity of Cu_2_O@C_2_N, we performed a descriptor-based analysis combining reaction energetics and electronic structure metrics. As shown in Figure 7a, the relationship between applied strain and methanol formation energy barrier shows an inverted volcano-type trend, with the lowest ΔG_overall_ (1.31 eV) at 1% tensile strain. This observation confirms that moderate strain yields an optimal electronic and geometric configuration for the Cu-O-Cu active center, thus enhancing reaction kinetics. To further rationalize this behavior, we conducted PDOS analysis and extracted the d-band center (ε_d_) of the Cu atoms and the p-band center (ε_p_) of the neighboring C/N atoms in C_2_N. As shown in Figure 7b, the electronic descriptor Δε = ε_d_ − ε_p_ correlates strongly with the ΔG for methanol formation. This descriptor captures the electronic coupling strength between the Cu active center and the support environment, reflecting how efficiently electrons can be transferred during the reaction.

At 1% tensile strain, methanol formation reaches the smallest energy span, as shown in Figure 7b, indicating the strongest synergistic interaction between Cu and the C/N support. Δε deviates from this optimal value when the strain is either too low (0%) or too high (≥3%), leading to unfavorable orbital overlap and higher activation energy barriers. These results coincide with the well-known Sabatier’s principle and emphasize that strain-regulated interfacial electron matching is crucial for maximizing catalytic efficiency. Collectively, this descriptor-based volcano trend not only explains the observed strain–activity relationship but also provides a predictive framework for screening and optimizing other metal–support combinations for selective methane oxidation. The Cu_2_O@C_2_N system thus exemplifies how strain-engineered d–p orbital alignment governs reactivity, offering new avenues for rational catalyst design. To confirm whether this electronic-level synergy leads to actual charge redistribution, we performed Bader charge analysis under 1% strain. The results reveal a significant charge transfer of ~0.81 e^−^ from Cu_2_@C_2_N to *O, indicating strong metal–adsorbate coupling. This enhanced charge reorganization confirms the physical origin of the Δε-driven synergistic interaction and aligns well with the pronounced catalytic improvement observed at this strain level.

Given the strong correlation between Δε and catalytic activity in the Cu-C_2_N system, it is reasonable to propose that this descriptor may also apply to other transition metals (e.g., Fe, Mn, Ni) supported on C_2_N or other 2D substrates. This potential transferability of Δε across different metal–support systems could facilitate more efficient catalyst screening and offer a broadly applicable electronic-structure-based design framework.

## 3. Computational Details

The spin-polarized density functional theory (DFT) calculations were performed using the Vienna Ab initio Simulation Package (VASP) [54,55]. The Perdew-Burke-Ernzerhof (PBE) functional is used to account for electron exchange-correlation effects [56], and the ion-electron interactions are described using the Projection Augmented Wave (PAW) pseudopotentials [57]. The van der Waals (vdW) interactions are treated using the DFT-D3 method [58]. The cutoff energy for plane waves is set to 400 eV, and the convergence criteria for the total energy and forces per atom are set to less than 10^−5^ eV and 0.02 eV·Å^−1^, respectively. A 2 × 2 × 1 supercell containing 48 carbon atoms and 24 nitrogen atoms is adopted to model the pristine C_2_N substrate. The Brillouin zone is sampled with a 2 × 2 × 1 k-points mesh, and PDOS calculations employed a denser 9 × 9 × 1 mesh. Additionally, a vacuum layer of 30 Å is applied in the z-direction to avoid spurious interactions between periodic images. In addition, we performed ab initio molecular dynamics (AIMD) simulations [59] under 500 K for 10 ps with a time step of 1 fs to examine the dynamical stability of the catalysts. Cl-NEB combined with improved dimer methods is employed to locate the transition states, which are verified from frequency analysis with only one image frequency [60,61,62].

The formula for the formation energy is indicated as follows:

$$E_{f}=E_{catal}-E_{c2n}-{2\times E}_{Cu}-E_{O}$$

where E_catal_ represents the total energy of the catalyst, including the C_2_N support and the active Cu_2_O moiety, E_c2n_ is the energy of a single layer C_2_N, E_Cu_ is the energy of a copper atom in the bulk phase, and E_O_ equals half the energy of an oxygen molecule. A negative E_f_ indicates thermodynamic stability.

The Gibbs free energy is obtained by the following formula:

ΔG=ΔE+ΔZPE−TΔS

where ΔE, ΔZPE and ΔS represent the changes in energy from DFT calculations, the zero-point energy, and the entropy, respectively. Methane oxidation reactions are typically studied in the temperature range of 125 to 200 °C. Therefore, we selected 200 °C, as it represents the upper limit of the relevant experimental range, and used it for the Gibbs free energy calculations to ensure better comparability with previous studies [63].

## 4. Conclusions

In summary, we have proposed a dinuclear Cu_2_O cluster embedded in a nitrogen-rich two-dimensional C_2_N framework as an effective bioinspired catalyst for methane partial oxidation. The Cu-O-Cu active site shows strong anchoring and electronic interaction with the C_2_N substrate, ensuring structural stability and redox tunability. Among the two investigated reaction pathways, the concerted mechanism is kinetically more favorable due to lower activation energies and stable intermediate binding. Strain engineering proves to be a powerful strategy for enhancing catalytic activity and pathway selectivity. Moderate tensile strain (especially at 1%) optimally tunes the Cu-O-Cu bond geometry and d-band center alignment, significantly reducing the ΔG_overall_. An inverted volcano-type trend is established between applied strain and catalytic performance, supported by both energetic and electronic descriptors. Furthermore, the strain-responsive regeneration of the active site by N_2_O illustrates the broader role of mechanical modulation in the catalytic cycle. This study not only demonstrates Cu_2_O@C_2_N as a promising catalyst for selective methane oxidation but also provides a generalizable framework for designing strain-tunable heterogeneous catalysts based on bioinspired motifs and two-dimensional materials.

## Figures and Tables

**Table 1:** Summary of ΔGoverall (eV) for two pathways on Cu_2_O@C_2_N catalyst under strains from 4% to −2%.

Strain (%)	Concerted Pathway	Radical–Rebound Pathway
4	1.82	2.04
3	1.72	1.96
2	1.45	1.98
1	1.31	2.09
−1	1.73	1.98
−2	1.78	1.98

## Data Availability

Data are contained within the article and Supplementary Materials.

## Supplementary Materials

The following supporting information can be downloaded at https://www.mdpi.com/article/10.3390/molecules30153073/s1: Figure S1: Projected density of states (PDOS) plots of Cu_2_O@C_2_N under applied strains of −2% to −1% and 1% to 4%. Figure S2: Schematic illustration of charge density difference in the Cu_2_O@C_2_N heterostructure under applied strains ranging from −2% (compressive) to 4% (tensile). Figure S3: Formation energy change curves for Cu_2_O@C_2_N applying −2% to 4% strain. Figure S4: Total energy change for atomic molecular dynamics (AIMD) simulations of Cu_2_O@C_2_N at 500 K for a sustained 10 ps applied strain. Figure S5: Variation in bond-length during AIMD simulation at 500 K for 10 ps (a) 0% strain, (b) −2% strain, and (c) 4% strain. Figure S6: Schematic illustration of intermediate and transition state structures along the reaction pathway for methane partial oxidation to methanol under 4% tensile strain. Figure S7: Schematic illustration of intermediate and transition state structures along the reaction pathway for methane partial oxidation to methanol under 3% tensile strain. Figure S8: Schematic illustration of intermediate and transition state structures along the reaction pathway for methane partial oxidation to methanol under 2% tensile strain. Figure S9: Schematic illustration of intermediate and transition state structures along the reaction pathway for methane partial oxidation to methanol under 1% tensile strain. Figure S10: Schematic illustration of intermediate and transition state structures along the reaction pathway for methane partial oxidation to methanol under 1% compressive strain. Figure S11: Schematic illustration of intermediate and transition state structures along the reaction pathway for methane partial oxidation to methanol under 2% compressive strain. Figure S12: Comparison of the free energy barrier for methanol formation and N_2_O activation energy barrier at the Cu_2_O@C_2_N active site under different strain conditions.
