# Supplementary material for: Strain Engineering of Cu2O@C2N for Enhanced Methane-to-Methanol Conversion
Source: Molecules. 2025 Jul 23;30(15):3073. doi: 10.3390/molecules30153073 (PMC12348963; doi:10.3390/molecules30153073)
Supplement: Supplementary file 1 [file molecules-30-03073-s001.zip › molecules-3735948-supplementary.pdf]

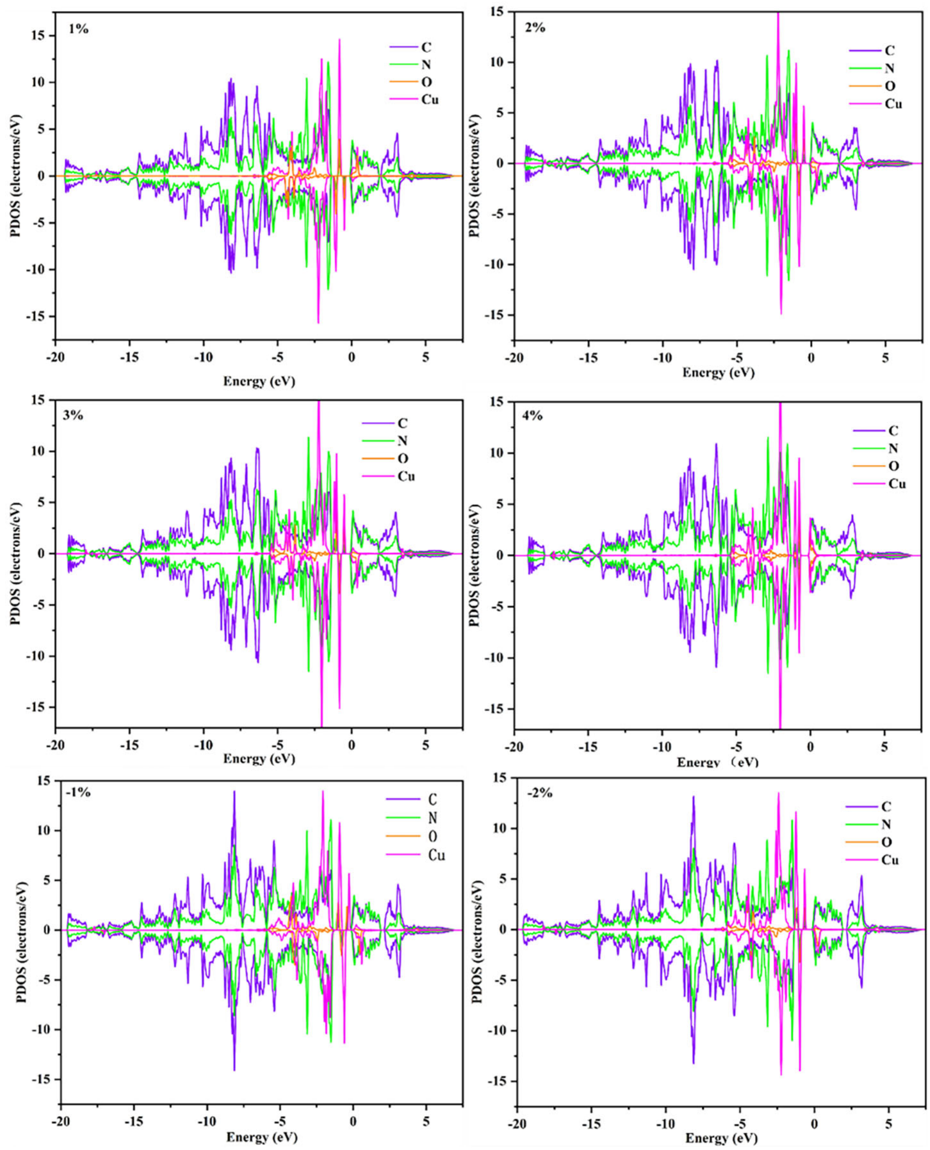

Figure S1. Projected density of states (PDOS) plots of $Cu_2O@C_2N$ under applied strains of -2% to -1% and 1% to 4%.

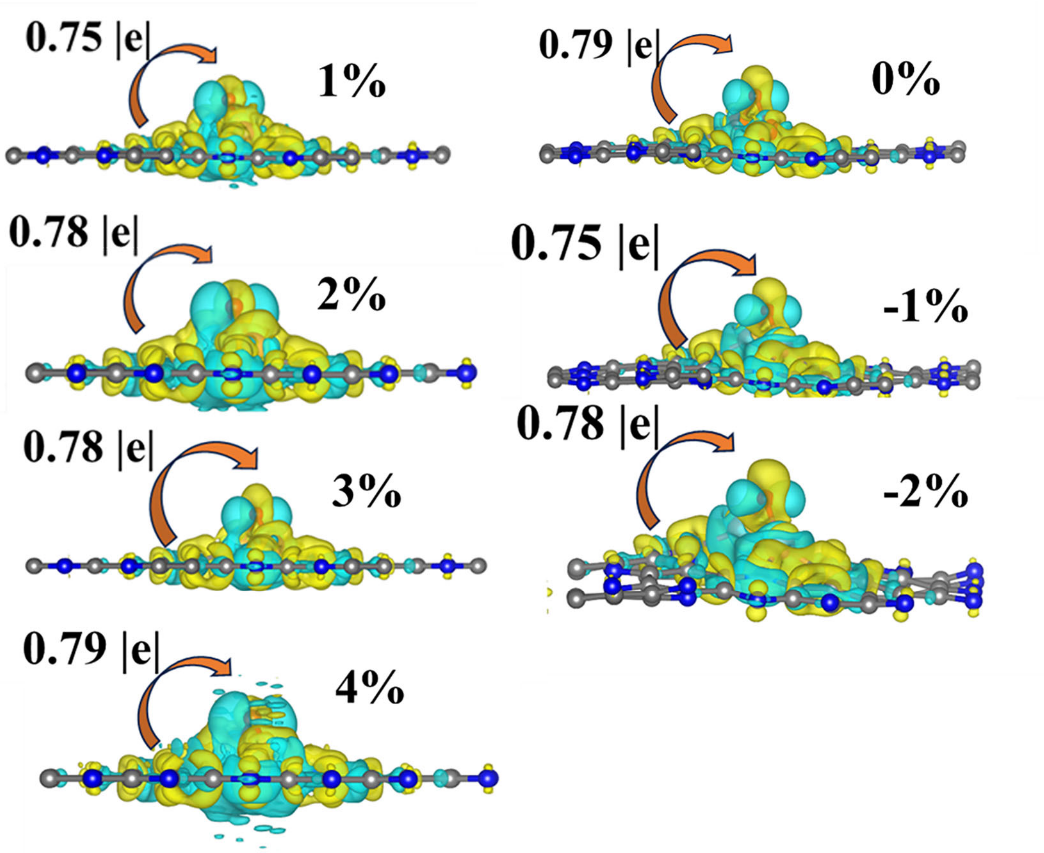

Figure S2. Schematic illustration of charge density difference in the $Cu_2O@C_2N$ heterostructure under applied strains ranging from -2% (compressive) to 4% (tensile).

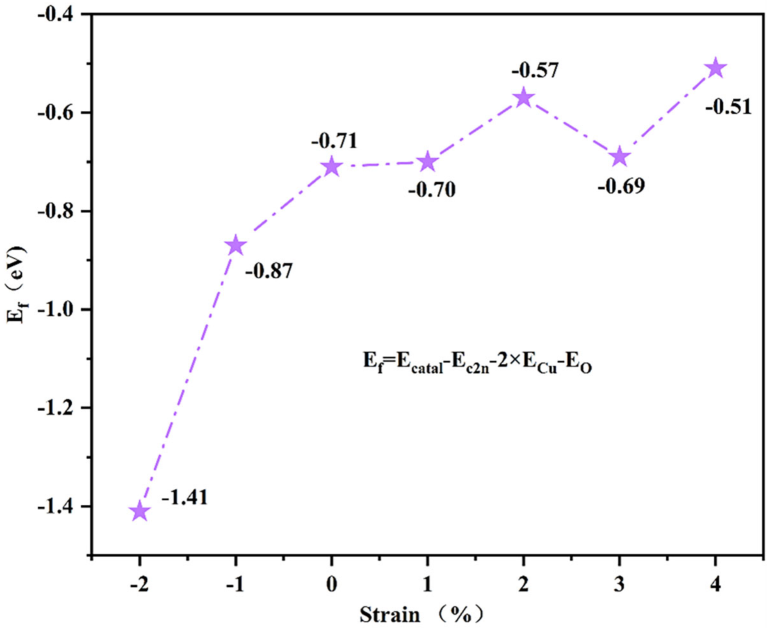

Figure S3. Formation energy change curves for $Cu_2O@C_2N$ applying -2% to 4% strain.

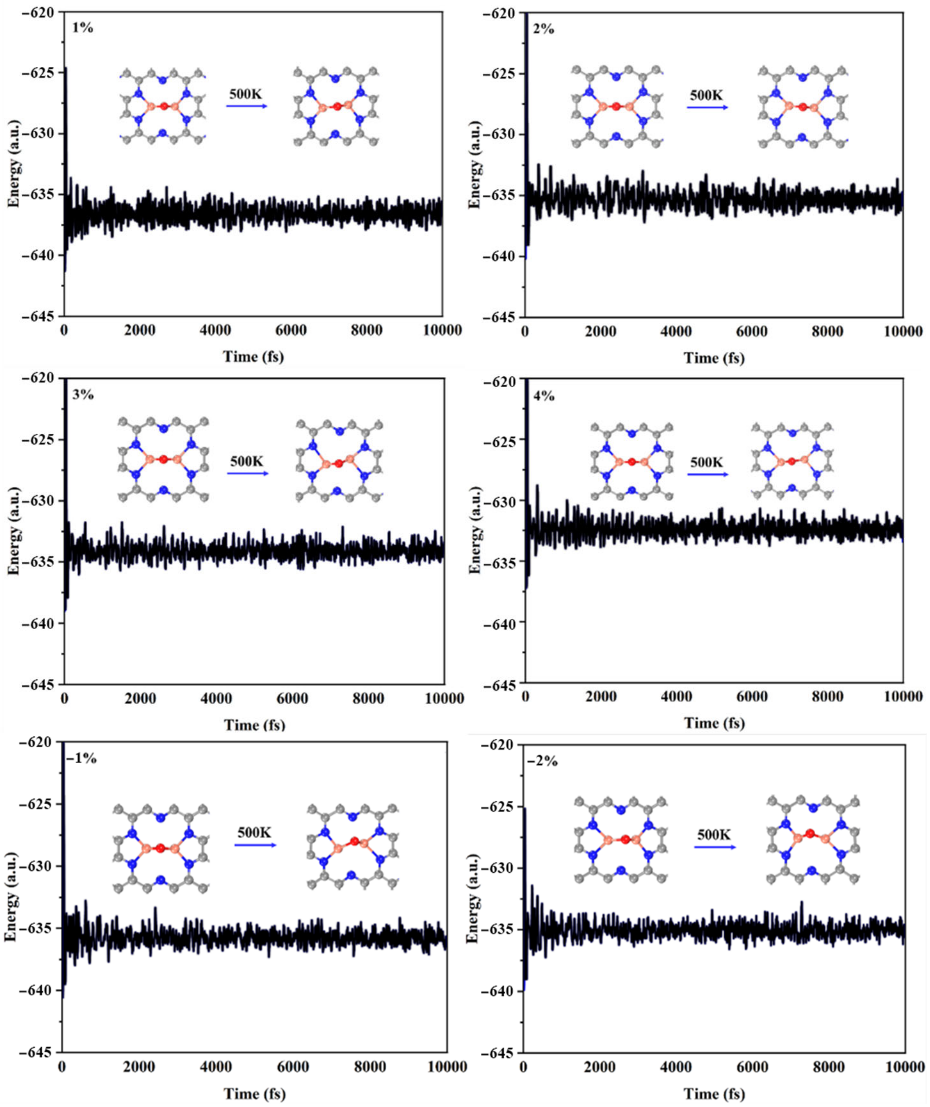

Figure S4. Total energy change for atomic molecular dynamics (AIMD) simulations of $Cu_2O@C_2N$ at 500 K for a sustained 10 ps applied strain.

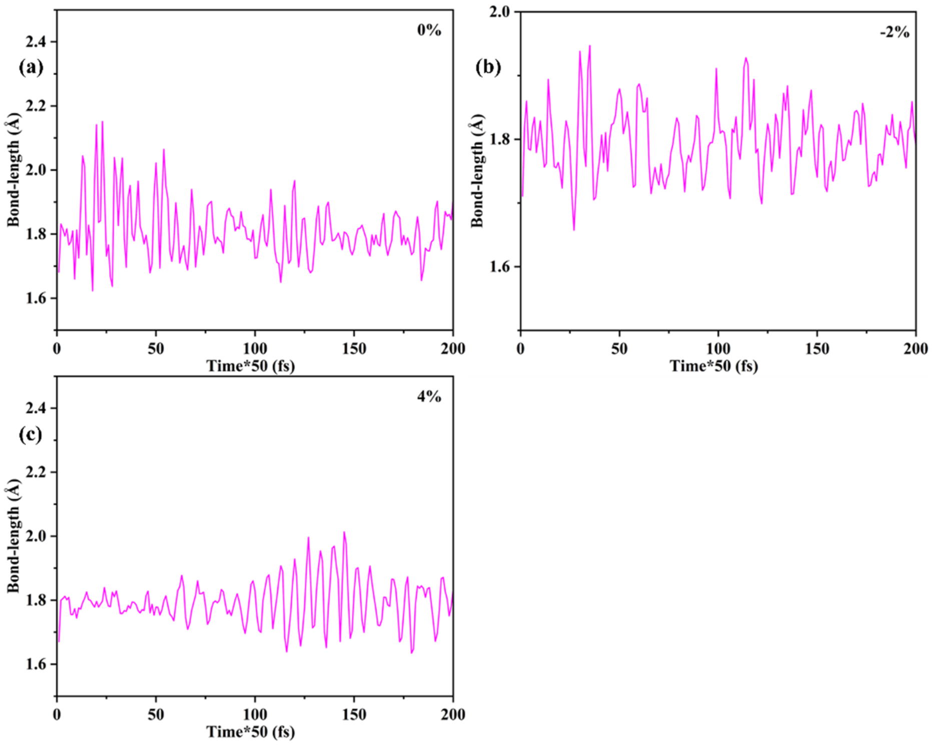

Figure S5. Variation of bond-length during AIMD simulation at 500 K for 10 ps (a) 0% strain (b) -2% strain and (c) 4% strain.

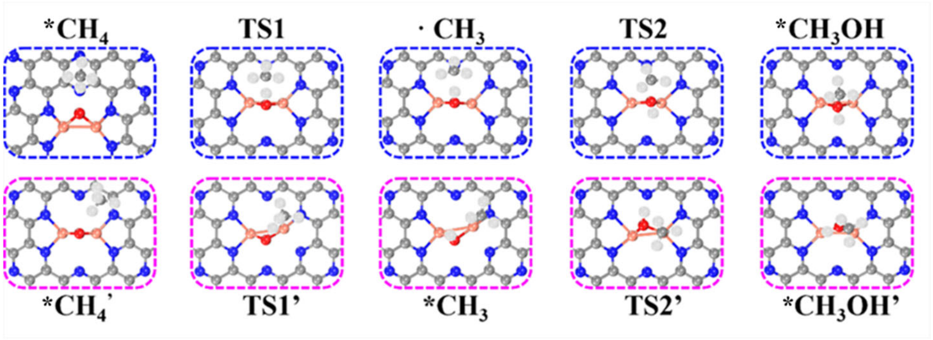

Figure S6. Schematic illustration of intermediate and transition state structures along the reaction pathway for methane partial oxidation to methanol under 4% tensile strain.

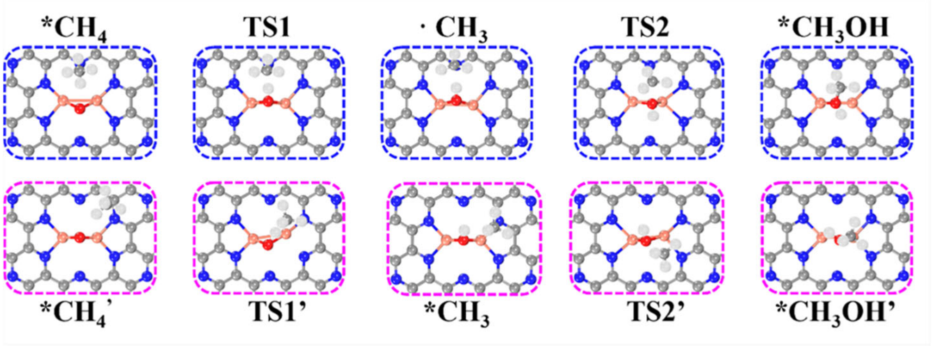

Figure S7. Schematic illustration of intermediate and transition state structures along the reaction pathway for methane partial oxidation to methanol under 3% tensile strain.

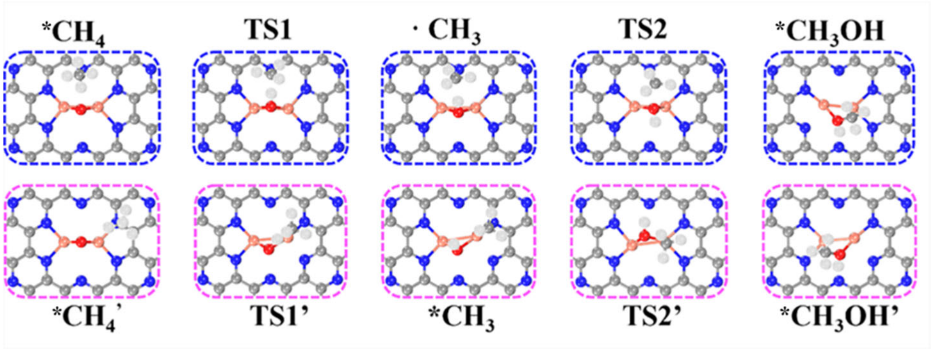

Figure S8. Schematic illustration of intermediate and transition state structures along the reaction pathway for methane partial oxidation to methanol under 2% tensile strain.

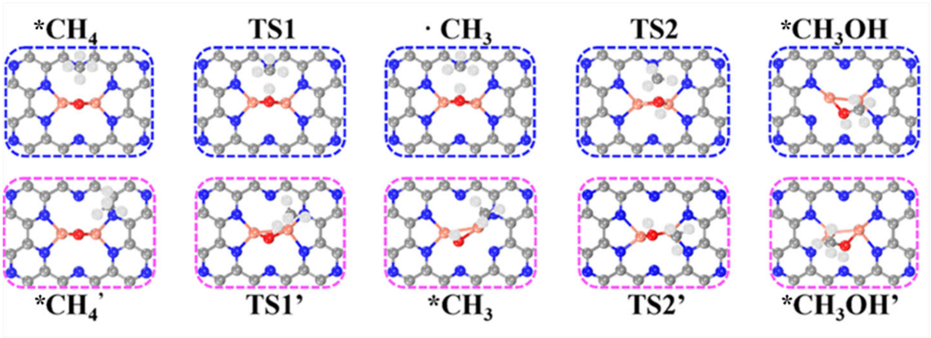

Figure S9. Schematic illustration of intermediate and transition state structures along the reaction pathway for methane partial oxidation to methanol under 1% tensile strain.

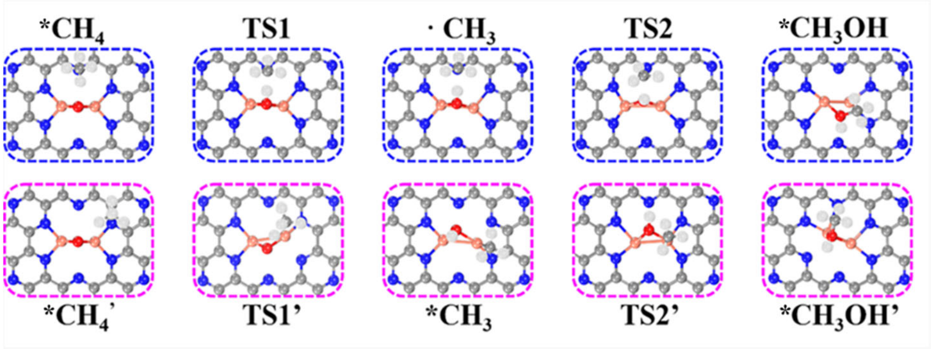

Figure S10. Schematic illustration of intermediate and transition state structures along the reaction pathway for methane partial oxidation to methanol under 1% compressive strain.

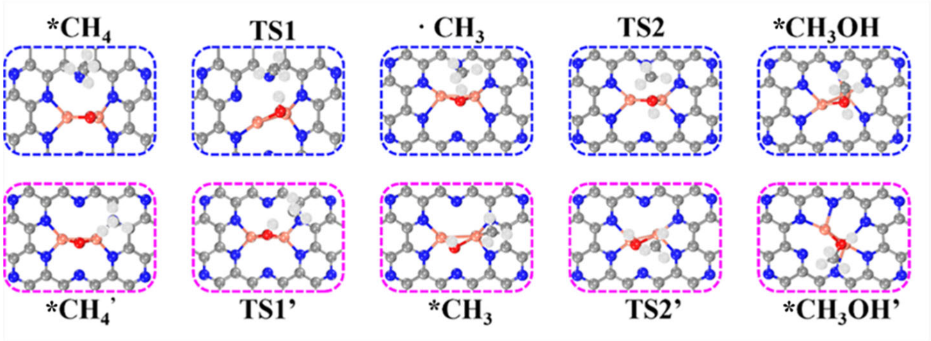

Figure S11. Schematic illustration of intermediate and transition state structures along the reaction pathway for methane partial oxidation to methanol under 2% compressive strain.

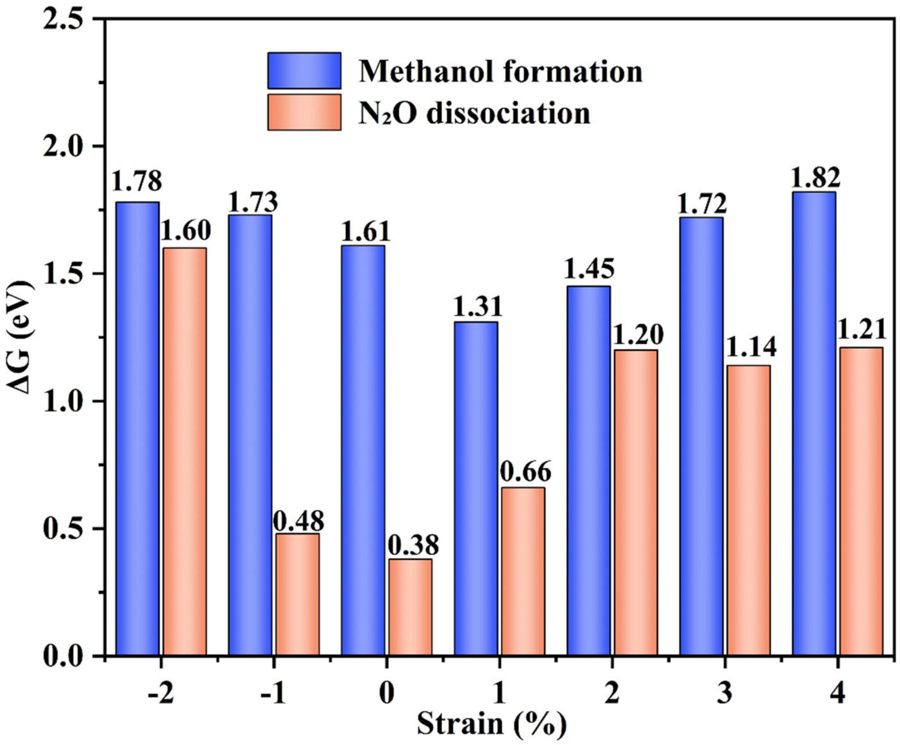

Figure S12. Comparison of the free energy barrier for methanol formation and N_2O activation energy barrier at the Cu_2O@C_2N active site under different strain conditions.
